# The MetaGens algorithm for metagenomic database lossy compression and subject alignment

**DOI:** 10.1093/database/baad053

**Published:** 2023-08-11

**Authors:** Gustavo Henrique Cervi, Cecilia Dias Flores, Claudia Elizabeth Thompson

**Affiliations:** Graduate Program in Health Sciences, Universidade Federal de Ciências da Saúde de Porto Alegre (UFCSPA), Rua Sarmento Leite, 245 - Centro Histórico, Porto Alegre, RS 90050-170, Brazil; Graduate Program in Health Sciences, Universidade Federal de Ciências da Saúde de Porto Alegre (UFCSPA), Rua Sarmento Leite, 245 - Centro Histórico, Porto Alegre, RS 90050-170, Brazil; Graduate Program in Health Sciences, Universidade Federal de Ciências da Saúde de Porto Alegre (UFCSPA), Rua Sarmento Leite, 245 - Centro Histórico, Porto Alegre, RS 90050-170, Brazil

## Abstract

The advancement of genetic sequencing techniques led to the production of a large volume of data. The extraction of genetic material from a sample is one of the early steps of the metagenomic study. With the evolution of the processes, the analysis of the sequenced data allowed the discovery of etiological agents and, by corollary, the diagnosis of infections. One of the biggest challenges of the technique is the huge volume of data generated with each new technology developed. To introduce an algorithm that may reduce the data volume, allowing faster DNA matching with the reference databases. Using techniques like lossy compression and substitution matrix, it is possible to match nucleotide sequences without losing the subject. This lossy compression explores the nature of DNA mutations, insertions and deletions and the possibility that different sequences are the same subject. The algorithm can reduce the overall size of the database to 15% of the original size. Depending on parameters, it may reduce up to 5% of the original size. Although is the same as the other platforms, the match algorithm is more sensible because it ignores the transitions and transversions, resulting in a faster way to obtain the diagnostic results. The first experiment results in an increase in speed 10 times faster than Blast while maintaining high sensitivity. This performance gain can be extended by combining other techniques already used in other studies, such as hash tables.

**Database URL** https://github.com/ghc4/metagens

## Introduction

Metagenomics is the processing and analysis of genetic material extracted from an environment (soil, fluids, water and others) through the DNA/RNA sequencing of the collected biological material (1). A huge volume of data is generated from modern sequencing machines, which can go from gigabytes to terabytes of data (2). Data analysis is a major challenge that involves a great demand for computer equipment and is carried out through specialized software that analyzes the data and produce results, including statistical data (3). While the genomic analysis is focused on a single biological subject, metagenomics collects genetic information from all biological subjects present in the sample (4). The deoxyribonucleic acid (abbreviated as DNA) is made of two linked strands forming a double helix, where each strand has a backbone of sugar (deoxyribose) with one of the four bases (adenine, cytosine, guanine or thymine) attached and phosphate groups (molecular biology). The amount of genetic material from a single DNA molecule is too small to be sequenced. The genetic material obtained from samples must be first duplicated and amplified. The first and Next Generation Sequencing (NGS) use a method called polymerase chain reaction (PCR), which is widely used and consists of thermal cycles that denatures (breaks) the DNA allowing the duplication and amplification processes (4–7). In the next phase, a machine identifies the nucleotide sequences. NGS (8) works in a massive parallel way, obtaining gigabytes of DNA data per run (chemical process cycle). Until the mid-2000s, when the first NGS sequencers appeared, it was hard to obtain data. Today, it is hard to analyze the huge amount of data (4).

Once biological agents can be detected from a fluid sample, the metagenomic technique eventually evolved into what is called clinical metagenomics (5), which has the purpose of identifying the etiological agents responsible for diseases allowing a fast diagnosis. A special chapter of this history is to use metagenomics as a tool for the diagnosis of infectious diseases (5–7). As an example, central nervous system (CNS) infections are commonly confused with other diseases and there are a variety of etiologic agents that are not easily identifiable. There are estimates that the identification of the etiologic agent occurs in only 25% of cases, even considering excellent laboratory facilities (9). These infections are neurological emergencies and have high morbidity and mortality. Consequently, it is essential that epidemiological studies be carried out and new diagnostic methods and therapeutic strategies be developed to reduce costs for the health system. Community-acquired CNS infections have an unfavorable outcome in ∼30% of cases, including severe sequelae or even death due to difficulty in identifying the etiologic agent and, consequently, leading to an inadequate treatment (10).

Considering the impact of metagenomics in helping to diagnose severe diseases and more rapidly initiate adequate treatment, as well as the importance of development of new and faster computational methods for analyzing the huge amount of data generated by metagenomics, this study aims to present a novel algorithm to optimize the database and process data to obtain faster results.

### Pipeline

**Figure 1.**
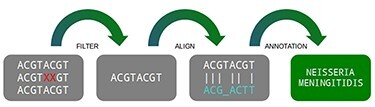
Three main steps of a metagenomic pipeline: filtering, aligning and annotating. Source: the authors.

A traditional pipeline has several steps chained together, each one containing its specific function. In general terms, a metagenomic pipeline can be divided into four steps: (i) filtering data, (ii) aligning with databases, (iii) filtering results and (iv) statistical reports (Figure 1). Despite the fact the DNA vocabulary is very reduced, containing only four letters (A, C, T and G), the combination of them are the “source code” of all living matter, which varies from a simple thousand-base bacteria to a multi-billion-base animal genome (4).

**Figure 2.**
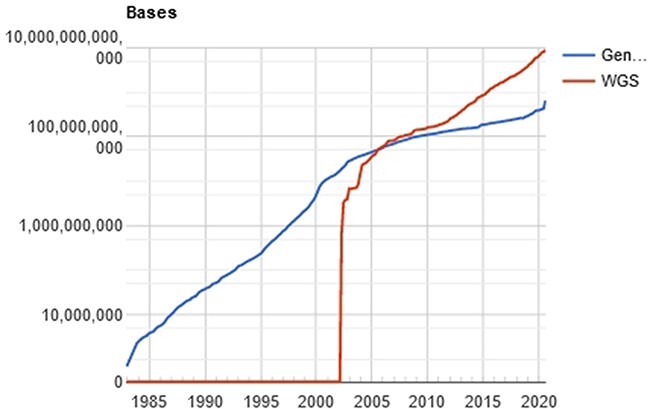
Evolution of the GenBank database, maintained by the NCBI. showing an increase in the curve of genomic data deposits, which results in computational difficulties of data processing. Source: NBCI statistics webpage.

The computational processing behind this amount of data is a challenging problem (11), especially when the researcher/physician is running against the time, for example, waiting for the diagnosis of a disease. In a computational perspective, the main time-consuming problem relies on the huge volume of data to be filtered, organized and compared. Once the genetic sequencer finishes the sequencing process, all data must be processed. The NGS includes technologies of second and third generation that produces a large number of “short reads,” in contrast to the first-generation sequencing based on the traditional Sanger method. Illumina NGS commonly produces sequences up to 300 (this value refers to the Illumina MiSeq; however, the Pacbio Sequencers can produce >30 000 bases per read using long-read technologies) nucleotide bases (adenosine, guanine, thymine or cytosine), represented as a string of data like “ACGGATCGATTCGATTG…”. The comparison of these sequences with the reference database results in a possible diagnosis with the identification of the specific etiological agent (virus, bacteria, and/or fungus). The reference databases are accessible from public sites like GenBank (NCBI, USA), EMBL Bank (EMBL-EBI, Europe) and DDBJ Bank (DDBJ, Japan), which easily overpass the terabyte of data. Figure 2 shows the evolution of the GenBank database through the years.

### Quality control

**Figure 3.**
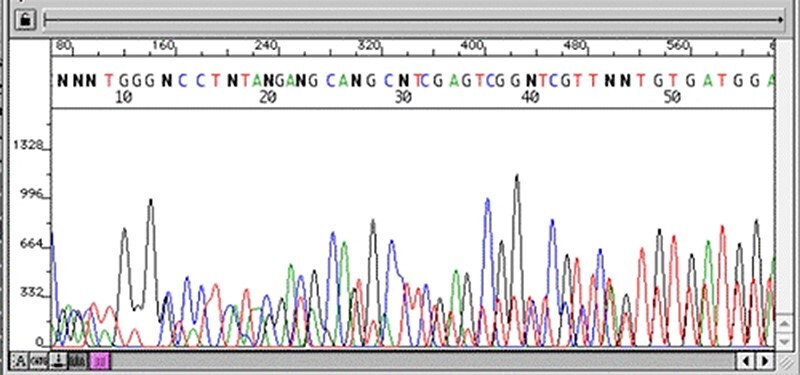
Chromatogram with multiple peaks per base—low quality data. Source: Roswell Park Comprehensive Cancer Center.

**Figure 4.**
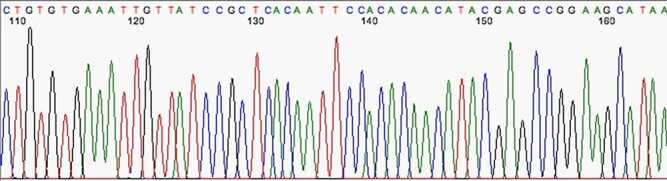
Chromatogram indicating good quality of data sequence. Source: U-M Biomedical Research Core Facilities.

The first stage after sequencing is the “quality control.” The sequencer produces a large amount of data, but not all with the same quality. This step is important to remove “low quality” data (12, 13). In summary, the first generation and most second generation sequencers are based on sequencing by synthesis, where information is collected through a fluorescent agent bound to the nucleotide (8), using a very precise wavelength laser, capturing the light emitted by the molecule to infer the sequence. The resulting signal may be biased or not deterministic. The sequencer calculates the “quality” of the read based on the light intensity and writes the score in the result file—each sequenced nucleotide has its specific quality score (14). Figures 3 and 4 show chromatograms with low (multiple peaks per base) and good quality data, respectively, obtained by a first generation sequencer (13).

### Amplification

All first and second generation sequencers require a DNA amplification step, where DNA is duplicated through PCR into multi-million copies, amplifying the signal to be detected by the machine. After sequencing, duplicate reads are removed resulting in data reduction (15).

### Host removal

The host removal, while is not mandatory for water and soil analysis, it is an important step for human diagnosis, speeding up the process and reduce the risk of bias. Since the sample is obtained from a living host (human), it is likely to have the host DNA present in the data after sequencing. This stage is performed by searching for the host DNA in the result file through comparison with the reference genome available in public databases. Once the host reads are identified, they must be removed. In case of human samples, the most recent and curated reference human genome is preferentially used.

### Searching through reference databases

**Figure 5.**
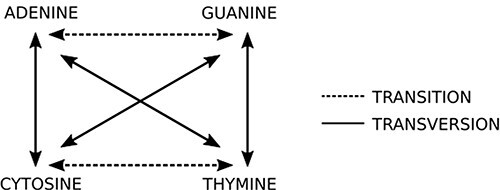
Two basic types of mutations: in transitions there is an exchange of bases of the same class (purine or pyrimidine), while in transversions there is an exchange of bases of different classes. Source: the authors.

This step is the most computing expensive task. The sequencer yields a huge amount (>100 million) of short reads (up to 300 nucleotides bases in current second generation sequencing) written in a text file, whose format is commonly the FASTQ type (14) (same from the original FASTA file format but with quality information). Each read is represented by one DNA string like “ACGATCGATTCGGA(…)” and it must be compared to reference datasets (terabytes of genomes from all sorts of living organisms, available on public organized databases). The first guess is O(m + n) like Knuth–Morris–Pratt or O(m)+ Ω(n/m) like Boyer–Moore algorithms could be applied to solve this problem. However, these algorithms are not able to produce efficient results from a biological viewpoint. To compare the new sequence to all sequences available in databases, it is necessary to perform sequence alignment. The DNA is not a rigid and static sequence, it is submitted to evolutionary forces, such as mutation, selection, genetic drift and migration. Considering the mutational aspect, the DNA substitutions can be classified as (i) transitions: when involve bases with similar chemical structure, interchanges of two-ring purines (A ⇋ G) or one-ring pyrimidines (C ⇋ T) and (ii) transversions: when involve substitutions of one-ring and two-ring DNA bases, interchanges of purine for pyrimidine bases and vice-versa. Figure 5 indicates the possible transitions and transversions.

**Figure 6.**
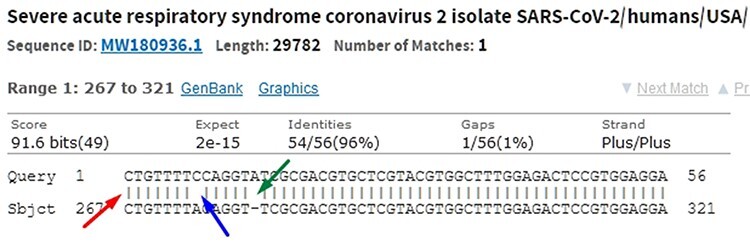
In the alignment of genetic sequences, mismatches (blue), indels (green) and matches (red) can occur. The occurrence of these events does not necessarily mean that they are different subjects. Source: the authors (sample on NCBI Blast).

When comparing two sequences to obtain an alignment, the main objective is to identify the positional homology, i.e. identify sites with a common ancestry in the alignment. It may be necessary to include gaps (indels, corresponding to deletion in sequence 1 and insertion in sequence 2) to better accommodate one sequence in relation to another. In this sense, the sequence ‘ACGATCGAT’ may be biologically equivalent to the sequence ‘ACGCTCGGAT’ (one mutation and one indel), i.e. they may be homologous. Homology is a biological concept that indicates two sequences share a common ancestry. Common algorithms used to align sequences in genomic research are Levenshtein Distance (16), Smith–Waterman (17), Needleman–Wunsch (18), Burrows–Wheeler (19) plus hashing and its derivatives. Blast, which uses a heuristic method based on Smith–Waterman, is the most used software to perform local alignment. It allows identifying subject sequences in a database that are like a query sequence. Figure 6 shows a local alignment obtained by a Blast search, with the indication of mismatches (blue arrow and lack of| symbol), indels (green arrow and gaps) and matches (red arrow and| symbol). It is important to note that another source of mismatches is associated with sequencer read errors.

### Dynamic programming

**Figure 7.**
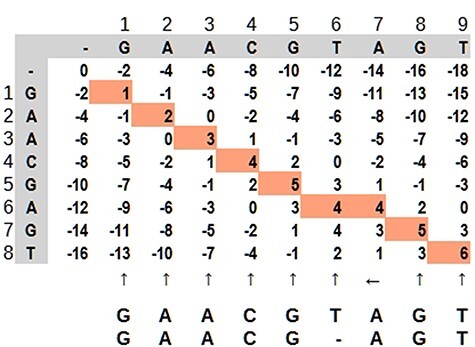
Dynamic programming illustrated by a matrix ‘mn,’ where the highlighted path represents the optimal alignment between the sequences. Source: the authors, based on (25).

Dynamic programming applied to bioinformatics (e.g. Levenshtein Distance, Smith–Waterman and Needleman–Wunsch) has complexity in order of O(mn) in the worst case, but it is possible to improve as demonstrated by Berghel and Roach (20). It is very time-consuming in terms of computational processing, although it is possible to parallelize the task since an input does not depend on other data in the same processing stage. It is not rare that a software solution has more than one combination of algorithms For example, in case of seed-and-extend algorithms, it is very common for a software aligner to use the Burrows–Wheeler algorithm to reduce data size and hash tables to find the seed portions. Dynamic programming applied to sequence alignment can be explained using a 2-dimensional matrix where two sequences are compared. There are three main steps: (i) matrix initialization; (ii) matrix fill (scoring) and (iii) traceback (alignment). Match, penalty-gap and mismatch values are defined according to a score (21). During the matrix fill, for each cell, all possibilities are evaluated and received a value: (i) in diagonal: match or mismatch; (ii) gap in sequence *y* and (iii) gap in sequence *x*. The traceback step determines the actual alignment(s) that result in the maximum score. In Figure 7, the best alignment is shown in red.

### Proposed algorithm

**Figure 8.**
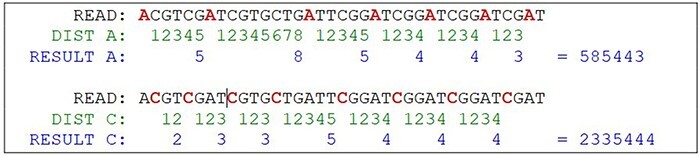
Hypothetical reads. The bases in red are used as a distance marker (green line). The result, in blue, makes up the sequence identity. Source: the authors.

To be feasible, the nucleotide matching algorithm must respect and reflect the nature of the DNA strand. In other words, the matching string must be straight enough to pair the sequence and be flexible enough to address the mutations and indels. The strategy behind this proposed algorithm is to divide and conquer, removing the most obvious non-matches from the database, limiting the fine search to the relevant subjects. To achieve this purpose, the algorithm computes the sequence to create identities of the reads, counting the distance between nucleotides A to next A and C to next C, simulating a wave where the same nucleotide interval is interpreted as a computed frequency (Figure 8). The cycle count obtained from the interval is stored in a database and used to perform the preliminary filter.

**Figure 9.**
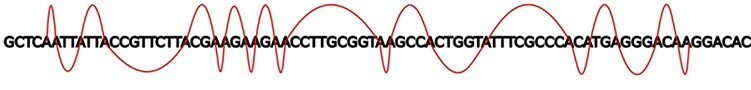
Example of a possible wave and its frequency. SARS-CoV-2 reference sample read from NCBI Sequence Read Archive (SAR): ERR4329467.

The algorithm locates the first nucleotide A in the sequence, then counts the number of nucleotides for the next A, and then repeats with the next A until the end of the read (Figure 9). The same process is performed with C nucleotide. The result of this computation is a set of numeric values containing the nucleotide distances, which is more efficient to evaluate than alphabetic values, such as “ACGT” in a computational approach. This algorithm reduces the size of the data by a fraction of the original. Mismatches on nucleotides G and T are irrelevant to the result, although it can be also calculated using the same method. Another important decision is that the repeating zones may be irrelevant because distances longer than nine are ignored (low priority) and distances equal to zero are ignored as well. These identities are used to match the reads to the reference genome in a highly sensitive way, discarding the unmatched reads due to the probability of a high *e*-value in a subsequent alignment. With the set of identities in a database, the next step is to filter and find possible matches with the reference genome, which must go through the same identity processing. The resulting data set is the block that will be used for the actual alignment of sequences, using consolidated techniques, such as the Blast algorithm.

**Figure 10.**
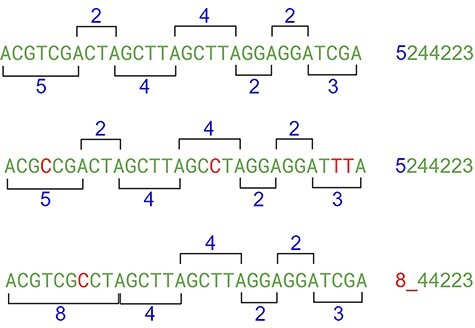
The indel problem. Source: the authors.

This approach may have flaws because of indels, where nucleotides can be inserted or deleted in the strain (Figure 10). In this case, the distance between the same nucleotides will vary. The solution is to create a range of tolerance using a substitution matrix where a distance between 19 and 20 (or a long range) is represented by the letter “A” (this parameter can be modified on the algorithm). Another approach relies on slicing the result code to reduce the final size of the comparison (Figure 10).

**Figure 11.**
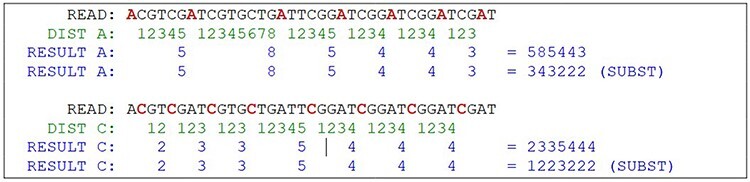
Partial result of the algorithm. Source: the authors.

After the range code substitution, the resulting sequence is “normalized” creating a kind of wave softness allowing the matching of sequences with small gaps or insertions (Figure 11).

The exact strategy to handle gaps is the substitution in Table 1 and the repetitions can be compressed using the sample in Table 2.

**Table 1.** Handle gaps substitution matrix. Source: the authors

Range (including)	Identifier	Range (including)	Identifier	Range (including)	Identifier
0.2	1	15.16	8	29.30	F
3.4	2	17.18	9	31.32	G
5.6	3	19.20	A	33.34	H
7.8	4	21.22	B	35.36	I
9.10	5	23.24	C	37.38	J
11.12	6	25.26	D	39.9999	Z
13.14	7	27.28	E		

**Table 2.** Naive compression substitution matrix. The X represents the repeating same gap size. Source: the authors

Repetition	Identifier
XXXXXXXXXX	a
XXXXXXXXX	b
XXXXXXXX	c
XXXXXXX	d
XXXXXX	e
XXXXX	f
XXXX	g
XXX	h
XX	i

## Experiments

**Figure 12.**
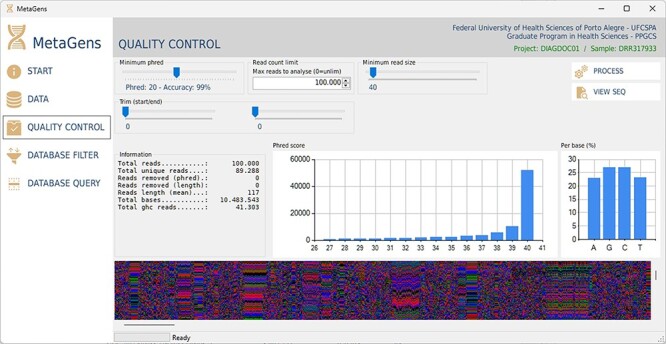
Graphical interface of the MetaGens software showing the quality control panel. It is a user-friendly interface that allows the specification of the quality control parameters. Source: the authors.

For the experiments, a tool called MetaGens with a graphical interface was built as an application for Windows (it can be ported to other operating systems). The purpose is to allow the researcher to build experiments with minimal computer knowledge. This tool allows the user to organize their research projects (diagnoses) into patients and runs. It is also possible to automatically download readymade experiments directly from NCBI and manipulate the algorithm parameters to vary its sensitivity and specificity. Figure 12 shows a screenshot of the quality control panel of a specific run performed by MetaGens, allowing the user to manipulate the controls and apply different filters.

**Figure 13.**
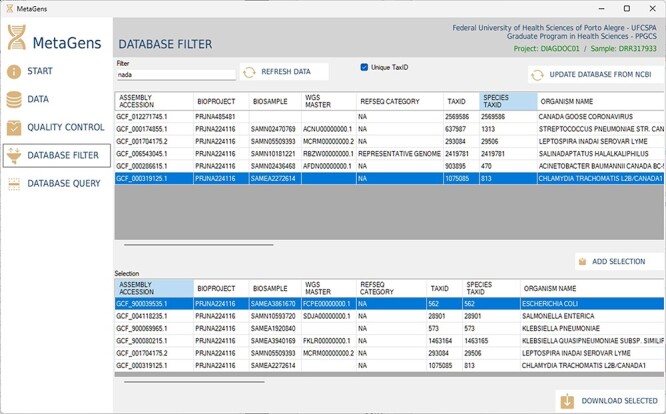
Graphical interface of the MetaGens software showing the database filter panel. When filtering the reference sequences, it is possible to reduce the size of the database in order to reduce the analysis time. Source: the authors.

Another important part of the tool is the selection of refseqs for building the diagnostic project. The researcher can filter references among the >220 000 records available in the NCBI database and further speed up the alignment process. This step is totally visual and the download of refseqs is also automated, directly from one of the available NCBI mirrors (cloud included). Figure 13 shows the database filter panel, which allows the reference selection.

**Figure 14.**
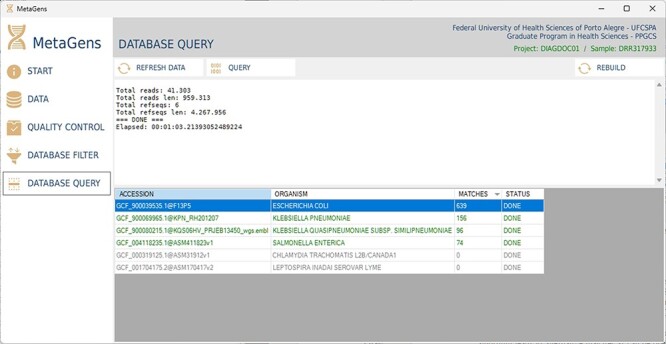
Graphical interface of the MetaGens software showing the database query panel. On the query screen, it is possible to follow the progress of the alignment. The table shows the subjects under analysis and their matches. Source: the authors.

After filtering and selecting refseqs, the next step is to process the sequences and align the reads with the selected refseqs. At this stage, the massive processing is computed. All CPU cores (CPU-threads included) are used in order to optimize the analysis. Figure 14 shows the database query panel, which is an alignment control panel where is possible to visualize some results that identify the etiological agents responsible for disease.

## Results and discussion

The first impact of the proposed algorithm (O(n)) is the data size reduction (∼80%) in comparison with the original nucleotide sequence. As an example, a sample read of SARS-CoV-2 containing 480 nucleotides yields a 79-character identity. The overall reduced size increases the main performance of the massive matching process. The algorithm is ∼10× faster than naive search (up to O(mn), both implemented in C#, using dotnet framework). As another example, the *Neisseria meningitidis* strain 433_NMEN (ASM106340v1) contains 2,397,512 bytes (nucleotides) before processing and 373,439 bytes after compression. The high sensitivity nature of the algorithm leads to alternative matches as can be observed on the NCBI SRA accession SRR12665177. The overall coverage leads to *N. meningitidis* (100%), although other subjects can be also matched (*Klebsiella quasipneumoniae*—98% coverage). Table 3 shows an example of the length of the files before and after the process.

**Table 3.** Comparison showing the size file between before and after processing files. Source: the authors

File	Original size (bytes)	Reduced Size (bytes)	Shrink %
GCF_000003925.1@ASM392v1	5 631 514	749 257	87
GCF_000006945.2@ASM694v2	5 013 482	797 157	84
GCF_900087615.2@WHOM	2 255 268	352 126	84
GCF_006334535.1@ASM633453v1	2 159 924	331 985	85
GCF_000002825.2@ASM282v1	54 436 962	6 471 680	88
GCF_004115315.1@ASM411531v1	6 507 834	1 034 216	84
GCF_003290055.1@ASM329005v1	5 893 322	970 815	84

For the experiments, a GUI was implemented in the dotnet framework and published at https://github.com/ghc4/metagens. Since the algorithm is based on lossy compression, the data reduction may be translated as loss of information, but it is not. Both data from the sequencer machine and the REFSEQ sequence are processed using the same technique. As consequence, the comparison is between the same “genomic language”—translated to a kind of wave. The final comparison is between the same metrics and the match is preserved. Once the distance between the nucleotides can vary due to insertions and deletions, a substitution range can be defined by the user, making the indels irrelevant. The transitions and transversions may be also irrelevant because the algorithm is not analyzing all nucleotides between the “waves,” only the “wavelength” is important to this approach. In comparison with dynamic programming, this technique is very permissive, detecting a great variety of subjects due to the nature of the matching strategy. Depending on how wide the parameters are, it is possible to reach false positives. However, at this stage, this algorithm may be more useful as a pre-filter, allowing faster dynamic programming (with smaller databases) or even a faster Blast.

In a direct comparison with the alignment algorithm used in Blast, the run SRR12665147 (taken from the NCBI biosample SAMN16133045) aligned with the subject *N. meningitidis* (GCF_008330805.1_ASM833080v1) resulted in over a million matches in 609.64 s of processing. On the same equipment, the proposed algorithm resulted in 23 622 matches in 49 s. The reduction in the number of matches occurs because the database was compressed and similar sequences were grouped. Moreover, almost all reads had some duplicates in the Blast analysis, streamlining the process. It was observed that the processing time has been reduced by >90%. Despite the comparison using the same hardware (i7-6700/64GB RAM), the Blast implementation is made using C/C++, while this algorithm prototype was written in C#, so an even higher gain is expected in the analysis of the results with better performance implementations (C/C++, Rust, etc). In another direct comparison, the same run (SRR12665147) aligned with the genome of *Neisseria sicca* (GCF_017753665.1_ASM1775366v1) took 175.47 s to process 69 235 matches, while the proposed algorithm took 27.74 s. Despite the *N. sicca* genome being of almost same size as the *N. meningiditis* genome, the number of matches directly influences the Blast processing time, but does not have a significant impact on the processing of the proposed algorithm since what weighs more in this case is the complexity of nucleotide sequences. It is important to emphasize that this study considers the matches to be relevant for the purposes of diagnosing infection. Consequently, the important thing is to identify the etiological agent and not necessarily its phylogeny. Table 4 shows a simple benchmark that has the illustrative purpose of comparing the result of a query using a reduced database with more complex tools, often containing complete genome banks, therefore it should not be evaluated in a 1:1 ratio.

**Table 4.** Benchmark comparison on the alignment of SRR12665147 with *Neisseria meningiditis* and *Neisseria sicca* (with partial database slice). Source: the authors.

Classifier	*N. meningiditis* (matches)	*N. meningiditis* (time)	*N. sicca* (matches)	*N. sicca* (time)
Kaiju	226 054	1589 s	490	1589 s
Kraken2	458 504	80 s	1035	80 s
BLAST	Over one million	609 s	69 235	175 s
Centrifuge	245 417	89 s	95	89 s
MetaGens	12 461 (compressed)	30 s	430 (compressed)	27 s

It is important to note that the number of matches does not necessarily mean greater sensitivity or accuracy. Considering that the reference database and accession are compressed, it is expected that the number of matches will be less than the same uncompressed data, as lossy compression will end up generating equal strings that will be discarded during the quality control process. It is also important to note that the compressed database does not use all genomes available in other tools, but a similar result can be obtained with the use of hash tables and, consequently, with greater use of RAM. This study has the purpose of propose an algorithm according to the similarity between sequences, not considering, at this moment, the analysis of taxonomic selectivity and sensitivity.

The metagenomics applied to the diagnosis of diseases is an important ally in several specific cases. Once the etiologic agent is identified, specific treatment can be implemented and the patient’s chances of improvement are increased. In some cases, as in infectious diseases of the CNS, the time to diagnosis is decisive in the outcome of the clinical case. The faster it occurs, the greater the chances of cure. The combination of several matching acceleration algorithms can be the key to the efficiency in the search for etiological agents in a large mass of genetic data.

In the last few years, clinical metagenomics has jumped from ∼70 publications on PubMed in 2010 to ∼540 publications in 2019, probably as a result of the advances on computational methods and development of new sequencing technologies. While the mainstream factories are spreading genetic sequencers through the biotech laboratories, some companies are developing pocket sequencers (22) at a cost of ∼U$4 500 00 that produce up to 30 Gb of data. In the near future, it may allow the self-diagnosis of some diseases with effective confidence, thus it will result in a massive amount of data to process, turning the analysis even more challenging.

## Conclusion

In some preliminary experiments, with use of functional massive parallel processing, it was possible to observe an interesting gain of performance in comparison with the very same structure running standard algorithms, using the reduced mass of data, although it is not yet parametrized or precisely measured because of the different metrics involved. Through the years, the researchers are evolving their techniques to speed up the analysis process and produce results faster (23–25). Computer technology is also evolving, increasing speed and capacities in new processor generation. However, the genomic databases are also growing in an exponential way. Consequently, it is necessary for a faster solution able to deal with large amounts of data comparisons, enabling the use of clinical metagenomics as an important weapon against infections of difficult diagnosis and treatment.
